# Influence of Different Diets on the Degradation of Sulfasalazine by Colon Bacteria Determined Using MimiCol^3^

**DOI:** 10.3390/ph16081128

**Published:** 2023-08-10

**Authors:** Dariah-Sohreh Seradj, Regine Beeck, Annika Haase, Julius Krause, Philipp Schick, Werner Weitschies

**Affiliations:** Center of Drug Absorption and Transport, Department of Biopharmaceutics and Pharmaceutical Technology, University of Greifswald, Felix-Hausdorff-Str. 3, D-17489 Greifswald, Germany; dariah-sohreh.seradj@uni-greifswald.de (D.-S.S.); regine_beeck@web.de (R.B.); haaseann@gmx.de (A.H.); julius.krause@uni-greifswald.de (J.K.); philipp.schick@uni-greifswald.de (P.S.)

**Keywords:** MimiCol^3^, dynamic colon model, in vitro metabolization, colonic microbiota, diet, sulfasalazine

## Abstract

The microbiome of the colon is characterized by its great diversity. This varies not only intra- but also interindividually and is influenced by endogenous and exogenous factors, such as dietary and lifestyle factors. The aim of this work was to investigate the extent to which the degradation of the drug sulfasalazine is influenced by different microbiota. Therefore, the in vitro model MimiCol^3^ was used, which represents the physiological conditions of the ascending colon. In addition to a representative physiological volume, the pH value, redox potential and an anaerobic atmosphere are important to provide the bacteria with the best possible growth conditions. Stool samples were taken from three healthy subjects, comparing omnivorous, vegetarian and meat-rich diets, and cultured for 24 h. However, the nutrient medium used for cultivation led to the alignment of the bacterial composition of the microbiota. The previously observed differences between the diets could not be maintained. Nevertheless, the similar degradation of sulfasalazine was observed in all microbiota studied in MimiCol^3^. This makes MimiCol^3^ a suitable in vitro model for metabolism studies in the gut microbiome.

## 1. Introduction

Due to the variety of different microorganisms, the human intestine represents a complex ecosystem. The dominant bacterial species and their composition varies in the different sections of the gastrointestinal tract. In the colon, the bacterial load is highest at 10^11^–10^12^ colony forming units (CFU)/mL. In contrast, the bacterial density in the stomach and small intestine is in the range of 10^1^–10^3^ and 10^4^–10^7^, respectively [1]. 

Since 2010, the subfield of pharmacomicrobiomics has focused on the interactions between variations in the microbiome and drugs. Further advances in enteric physiology and microbiota are accompanied by an increasing interest in prophylaxis, therapy, diagnostics and thus also in the development of drugs and dosage forms [2]. Therefore, the representation of individual sections of the gastrointestinal tract in suitable in vitro models is inevitable. Due to the interindividual and intraindividual variability of the intestinal microbiota, the colon represents a particular challenge here. The taxonomic subdivision of the colon microbiome includes 6–7 phyla, dominated by the phyla *Bacteroidetes* and *Firmicutes*. Most of the colon bacteria live in an obligately anaerobic environment (>95%) [3]. Aerobes in the colon include, among others, *Escherichia coli* and eukaryotic microorganisms such as yeasts and fungi. These microorganisms are part of a dynamic system that is shaped by prevailing life circumstances. They are formed by genetic factors and influenced by aspects such as lifestyle, diet and the intake of drugs [4,5]. 

The main functions of bacteria are the digestion of food components, microbial modulation of toxicity, vitamin synthesis and the imprint of the intestinal immune system [6,7,8]. In addition to the formation of gaseous end products, the bacterial cleavage of indigestible food components also leads to the formation of short-chain fatty acids (SCFAs) [9]. The formation of these SCFAs mostly involves the genera *Bacteroides*, *Bifidobacteria* and *Clostridia* [10]. This leads to the lowering of the pH in the colon to a range of 5.5–6.5, making it more difficult for various pathogenic germs to accumulate in this region [8]. Nevertheless, certain dysbiotic changes in the composition of the microbiota can also have negative effects on the human organism. In terms of the complexity and composition of its microbiome, each individual is unique. There is thought to be a link between metabolic and inflammatory diseases and the microbiome. For example, an imbalance in the dominant phyla *Bacteroidetes* and *Firmicutes* can lead to various diseases such as obesity or intestinal inflammation [6]. Therefore, enterotypes are classified according to the dominant species. The first enterotype is associated with an increased occurrence of *Bacteroides*. Due to the saccharolytic potential of this genus, energy production appears to be largely from the fermentation of carbohydrates and proteins. The second enterotype is dominated by the genus *Prevotella*. Here, high levels of carbohydrates and monosaccharides are typically found. Finally, the *Ruminococcus*-enriched type is considered the most abundant. All three types use different means of energy production from the substances available in the colon [11,12]. This is indicative of an ability to adapt to the prevailing living conditions. In recent years, increasing attention has been focused on the different dietary habits of people. Thus, different bacterial compositions of the microbiota have been found between vegans, vegetarians and people who also consume animal-based products.

A decrease in fecal pH has been described with plant-based diets, which is due to the higher concentration of SCFAs from the cleavage of carbohydrates [13,14]. A high-fiber diet leads to the expansion of species diversity and an increase in the phylum of *Bacteroidetes*, especially the genus *Prevotella*. Studies have reported differences in the number of bacteria of the genus *Bacteroides*, which decreases over the course of a prolonged low-meat diet. In addition, lower levels of *Lactobacilli*, *Bifidobacteria* and *Enterobacteria* have been found in vegans and vegetarians compared to omnivorous controls [13,15,16]. The pronounced influence of diet on the microbiome is considered to effect pathological changes [12,13,14,15,17]. The concept of influencing the microbiome through the consumption of certain products is not new and demonstrates the potential, but also the difficulties, of using pro- and prebiotics [18].

As with food, drugs can also interact with the microbiome. These include antibiotics, whose influence on the intestinal flora is often considered to be negative [19]. By unbalancing the intestinal system, they can promote colonization with pathogenic strains of bacteria. Other groups of drugs that can influence the microbiome include laxatives and proton pump inhibitors [20]. On the other hand, these microbes can also influence the metabolism of drugs. These drugs can be altered in their structure, bioavailability, effects and side effects. This can lead to both reactivation/activation and toxification [20]. In circumstances where the microbiome has a positive impact, as mentioned above, the drug is converted to its active form through metabolization. For instance, the anti-inflammatory drug sulfasalazine contains an azo bond that is cleaved by bacterial *Azoreductases.* This results in two molecules, sulfapyridine and the active metabolite 5-aminosalicylic acid [21]. *Azoreductases* are mainly secreted by representatives of *Enterobacteria*, *Clostridia* and *Bacteroides*. The catalytic activity of these enzymes is associated with an anaerobic metabolic environment [22]. The reason for the sensitivity to molecular oxygen are the co-substrates NADH and NADPH, which act as electron donors. Under aerobic conditions, these are increasingly consumed during cellular respiration and are thus less available for the electron transfer of the cleavage reaction [23,24].

The investigation of the influence of the microbiota on drugs requires suitable in vitro models. The physiological and anatomical conditions of the colon are particularly challenging. One in vitro model, the simulator of the microbial ecosystem of the human intestine (SHIME) with its five vessels, represents the entire gastrointestinal tract from the stomach to the descending colon. In 1993, Molly et al. succeeded in using this model to, as realistically as possible, represent parameters such as pH, temperature, transit times and the addition of enzymes [25]. The SHIME can be extended by a mucosal simulator [26]. This simulates the microbial colonization of the mucosa. Another in vitro model of the colon is the TNO in vitro model of the colon (TIM-2), which is a further development of the TIM-1 system that previously represented the sections of the stomach and small intestine. The TNO TIM-2 consists of four glass compartments and can also represent the intestinal peristalsis by using a flexible membrane [27]. Another in vitro model, the MimiCol^3^, introduced by Beeck et al., represents only the ascending colon [28]. Here, it is possible to use a simplified setup to represent the physiological conditions. In brief, the model replicates the physiological conditions of the ascending colon. Continuous nitrogen gassing creates an anaerobic atmosphere for the added colonic bacteria, ensuring optimum growth conditions. The construction and use of a shaking water bath allows the contents of the three glass vessels to be mixed and tempered simultaneously. Critical parameters can be measured during the experiment using inserted electrodes and the pH value can be controlled using pumping systems. Therefore, the MimiCol^3^ offers a compromise between the static SHIME and the highly complex TIM-2, by mimicking the dynamic conditions in the ascending colon without being overly complex or expensive. 

Thus far, the MimiCol^3^ has only been used with one standardized microbiota from a single healthy volunteer. The aim of the present work was to use samples from three healthy volunteers following different dietary lifestyles. Using these different microbiota, the rate and extent to which sulfasalazine metabolism is affected by dietary changes in the microbiome was investigated.

## 2. Results

### 2.1. Cultivation and Characterization of Three Different Standard Microbiota

#### 2.1.1. Biostat^®^ A Plus Process Parameters

**Figure 1:**
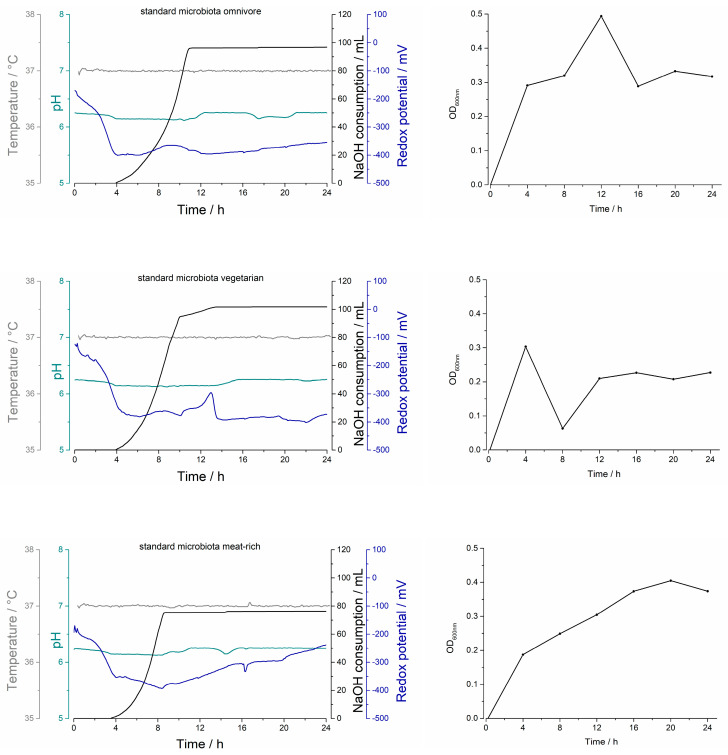
Process parameters (temperature (grey), pH (cyan), NaOH consumption (black), redox potential (blue)) and optical density in the Biostat^®^ A plus during the cultivation of three different standard microbiota over 24 h (2 L complex culture medium, 200 rpm, pH 6.2 (±0.1), 37 °C, *n* = 1).

Figure 1 shows the process parameters in the Biostat^®^ A plus during the cultivation of different standard microbiota from the healthy volunteers. 

The temperature remained stable during all experiments. Between 4 and 10 h, the exponential consumption of 1 M NaOH was noted in all three experiments. The consumption did not increase further after 10 h. The redox potential decreased rapidly in all three experiments within the first 4 h. While there were no more notable fluctuations in the SM_omni_ and the value leveled off at −350 mV, there was an increase between 10 and 12 h in the SM_veget_. Thereafter, this remained at a constant level. In the cultivation of the SM_meat_, the minimum was reached after 8 h with approximately—400 mV. During the experiment, a gradual increase in the redox potential could be observed. By the end of the 24 h of the experiment, the redox potential had increased to almost the same value as when the experiment was initiated.

#### 2.1.2. Optical Density

**Figure 2:**
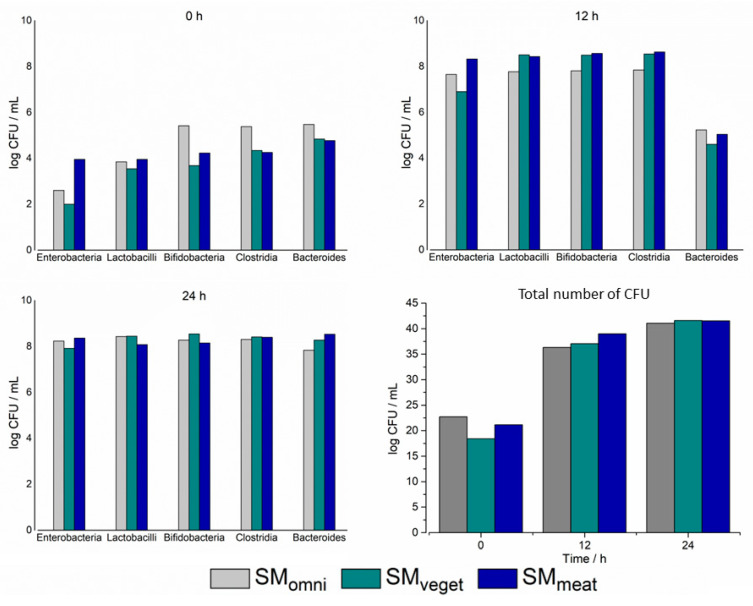
Bacterial composition of the complex bacterial culture over the cultivation period of 24 h. Representation of individual bacterial strains at the time points 0, 12 and 24 h and total number of CFU (Biostat^®^ A plus, 2 L complex culture medium, 200 rpm, pH 6.2 (±0.1), 37 °C, *n* = 1).

During cultivation, bacterial growth was monitored by optical density (OD) measurement. Figure 2 shows the progression of bacterial growth during the cultivation of the three different standard microbiota over 24 h. A distinct variation in bacterial growth could be observed during the cultivation process. For the SM_omni_, a steep increase occurred within the first 4 h, with the maximum OD being reached after 12 h. Subsequently, the absorption decreased again markedly until 16 h and remained constant towards the end of the experiment. The course of the SM_veget_ was different. During this experiment, the maximum OD was reached after 4 h, after which there was a significant drop in absorption until 8 h, followed again by an increase until 12 h. It then remained constant until the end of the cultivation. In the current study, the SM_meat_ showed completely different bacterial growth. The cell density had a slow but steady increase up to 20 h, and then slightly decreased in the last 4 h. 

Furthermore, Figure 1 illustrates the relationship between the consumption of the base and bacterial growth. Given that the specification for the experiments required that the pH value was maintained at 6.2 (±0.1) throughout the fermentation, the system counteracted the drop in pH by adding 1 M NaOH. In all three experiments, an increase in optical density absorption occurred within the first 4 h and the base supply was started. When growing the SM_veget_, the highest consumption was 120 mL. For both the SM_omni_ and SM_veget_, a plateau was reached after approximately 11 h. The SM_meat_ showed different results. The final volume of base addition was reached after 76 mL, with the difference from the SM_omni_ and SM_veget_ being the step increase at the beginning of the experiment.

#### 2.1.3. Characterization of the Three Different Standard Microbiota Using Selective Agar

In addition to determining the compositions of anaerobic and aerobic bacteria, the presence of *Enterobacteria*, *Lactobacilli*, *Bifidobacteria*, *Clostridia* and *Bacteroides* was also investigated. For the analysis, the composition of the sample was determined using the five bacterial sub-populations. However, no true value of the proportions was determined. Instead, the CFU of each strain were determined in relation to the entire CFU. In Figure 2, it can be seen how the concentrations of the five bacterial species changed over the 24 h cultivation period in the Biostat^®^ A plus.

The initial colonization of SM_omni_ was highest at this stage. This could be seen in the presence of *Bifidobacteria*, *Clostridia* and *Bacteroides*. In contrast, SM_veget_ had the lowest initial concentration. At time 0 h, the *Enterobacteria* represented the smallest share in all standard microbiota. However, over the entire course of the experiment, they had the most pronounced growth. In general, it can be stated that within the first 12 h, there was a marked increase in the concentrations of all five bacterial species. This remained at a similar level until the end of the experiment, except for the genus *Bacteroides*. This bacterial sub-population showed enrichment only in the second half of the experiment. After the end of the fermentation, the results of the products were quite consistent. The initial differences in bacterial concentration and composition were much less noticeable or non-existent.

To determine the anaerobic and aerobic bacteria, the Schaedler agar and TSA media were used. To calculate the percentages, the CFU of the anaerobes and aerobes were related to the sum of the two species.

**Figure 3:**
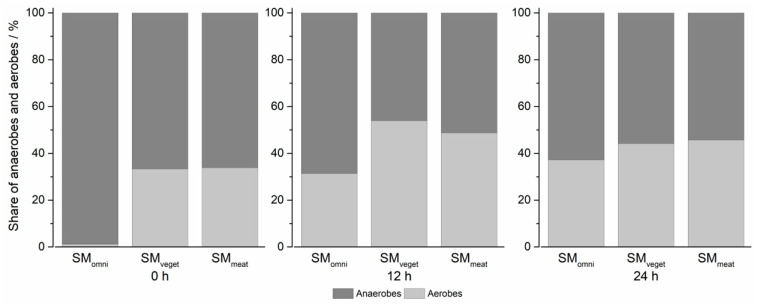
Share of anaerobes and aerobes in the complex bacterial culture over the cultivation period of 24 h (Biostat^®^ A plus, 2 L complex culture medium, 200 rpm, pH 6.2 (±0.1), 37 °C, *n* = 1).

Figure 3 shows the percentage composition over 24 h in the Biostat^®^ A plus during the cultivation of the standard microbiota. At time 0 h, anaerobes composed almost 100% of the SM_omni_. In the SM_veget_ and SM_meat_, the proportions were considerably lower at around 60%. After 12 h, there was a reduction in the anaerobic bacterial species in all standard microbiota. This proportion increased again slightly in the second half of the experiment. After a cultivation period of 24 h, the differences between the three standard microbiota were essentially non-existent. The individual standard microbiota was still composed of 50–60% anaerobic bacteria. The highest proportion of anaerobic bacteria was found in SM_omni_, which contained approximately 60%.

### 2.2. Investigation of Sulfasalazine Degradation and Bacterial Growth Using MimiCol^3^

#### 2.2.1. Process Parameters

After the addition of the standard microbiota at time 0 h, the redox potential decreased in all vessels. During the media changes at 3, 5 and 7 h, an increased redox potential and pH value could be observed. The addition of fresh Schaedler broth resulted in an increase in the pH value. At the media change, the temperature decreased by approximately 2 °C, even though the new medium was pre-tempered, and increased again shortly afterwards.

#### 2.2.2. Optical Density

**Figure 4:**
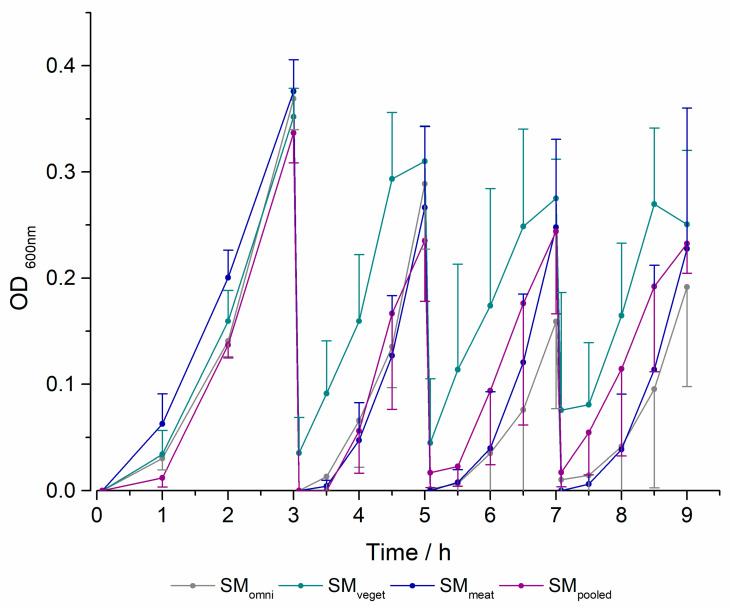
Optical density in the MimiCol^3^ over 9 h (150 mL Schaedler broth, 100 rpm, pH 6.2 (±0.25), 37 °C, *n* = 4 for SM_omni_, SM_veget_ and SM_meat_, *n* = 3 for SM_pooled_, (mean +/−SD)).

To screen bacterial growth during the experiment in the MimiCol^3^, the optical density was measured. Figure 4 shows the OD measurements of the standard microbiota used during all experiments. After an initial delay, there was a steep increase in absorption for all standard microbiota and the pooled microbiota up to the 3 h sample point. At this point in time, the highest measured OD for all standard microbiota was observed. After the first change in media, a noticeable drop could be noted. The SM_veget_ achieved the highest absorption and the fastest increase during the experiments, followed by the SM_pooled_. The renewed bacterial growth after the last two media changes was lowest in SM_omni_. Similar behavior could be seen between the SM_pooled_ and the SM_meat_. It can be said that the fluctuations around the mean value were marked in all bacterial cultures, and these increased substantially with the number of media changes.

#### 2.2.3. Degradation of Sulfasalazine

**Figure 5:**
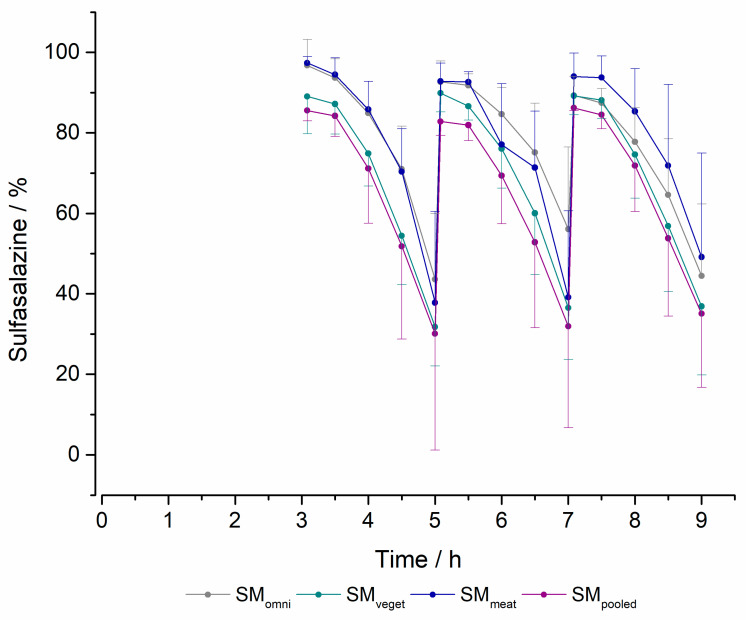
Metabolization of sulfasalazine in the MimiCol^3^ after a media change at 3, 5 and 7 h (150 mL Schaedler broth, 100 rpm, pH of 6.2 (±0.25), 37 °C, *n* = 4 for SM_omni_, SM_veget_ and SM_meat_, *n* = 3 for SM_pooled_, (mean +/− SD)).

In Figure 5, the results of the enzymatic sulfasalazine degradation for each microbiota are shown. During the media changes at 3, 5 and 7 h, sulfasalazine was added. Degradation of the drug occurred with all standard microbiota tested in the MimiCol^3^. However, differences could be observed between the different standard microbiota. Based on the standard deviations shown, there were noticeable differences in metabolization, particularly before the media changes and the end of the experiment. The metabolization of sulfasalazine tended to be lowest in SM_omni_, where the curves indicated a flattened decrease. The maximum drug degradation was 70%. This was seen in SM_veget_ and SM_meat_ as well as in SM_pooled_.

To be able to make a statement about the metabolization rates of the different bacterial cultures, the drug metabolism was determined within a given timeframe. Here, variations between the different standard microbiota could be found. While SM_meat_ recorded the highest metabolization in the first interval, this was observed for SM_veget_ in the second interval. In the SM_pooled_, a decrease in the metabolization rate was seen over time, whereas SM_omni_ metabolized sulfasalazine at the lowest rate.

#### 2.2.4. Determination of the Three Different Standard Microbiota and the Pooled Standard Microbiota Using Selective Agar

**Figure 6:**
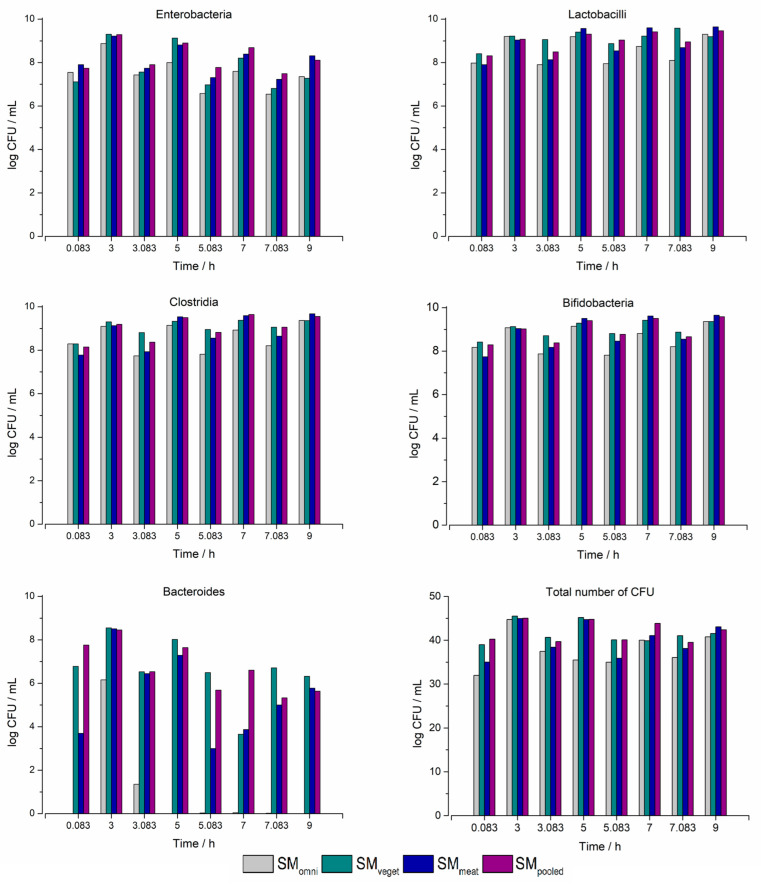
Bacterial composition of the different bacterial culture in the MimiCol^3^ and the total number of CFU over 9 h (150 mL Schaedler broth, 100 rpm, pH 6.2 (±0.25), 37 °C, *n* = 1).

As with the cultivation of the standard microbiota in the Biostat^®^ A plus, the characterization of the bacterial species was also carried out for the experiments in the MimiCol^3^. For this purpose, the CFU samples of the time points 0.083, 3, 5, 7 and 9 h, as well as 5 min after a media change, were characterized. Figure 6 shows the bacterial density of the five investigated bacterial species and the total number of CFU over the experimental period. For comparison, the samples from vessel 1 of each series of experiments were plated out and characterized. Except for the genus *Bacteroides*, an increase in bacterial growth was observed in all standard microbiota between the individual media changes. While the bacterial density of the genus *Enterobacteria* decreased over the experimental period of 9 h, it increased in the genus *Bifidobacteria*. Smaller differences could be noted for the *Lactobacilli* and *Clostridia*. For these bacteria, similar densities were reached after the first media change. The genus *Bacteroides* showed a different course. Here, significant differences could be noted in the individual intervals, as well as between the different standard microbiota. 

## 3. Discussion

The focus of the current work was on replicating the production of bacterial colonies and drug metabolism. Therefore, three different standard microbiota were investigated. Since the diets of humans have an influence on the gut microbiome, this was the main criterion, amongst others, used for the recruitment of different microbiota [29]. To be able to test the different standard microbiota in the MimiCol^3^, they were cultivated separately in the batch fermenter over a period of 24 h. Thereafter, the standard microbiota were characterized with regard to their species composition. To obtain an overview of the bacterial compositions of the different standard microbiota, CFU samples were plated out on agar media. To evaluate the cultivation of microbiota over the 24 h period, three samples were plated at 0, 12 and 24 h. Due to the options available at the time of the present study, the determination of the bacterial composition was performed using a single plating and the agar media described in Table 2. The data collected during the sampling process showed that the cultivation of the individual standard microbiota was successful—especially at the beginning of the cultivation in the Biostat^®^ A plus, where differences between SM_veget_ and SM_meat_ could be observed. For example, the high concentration of *Enterobacteria* in the SM_meat_ could be linked to the increased consumption of animal proteins [16]. Furthermore, the accumulation of primarily carbohydrate-splitting and thus acid-producing bacterial species could be observed in the SM_veget_ [14,30]. As can be seen from Figure 2, the bacterial species *Enterobacteria*, *Lactobacilli*, *Bifidobacteria* and *Clostridia* almost reached their maximum bacterial densities in all standard microbiota after 12 h. Hence, during the second half of the cultivation period, there was almost no increase in bacterial growth. However, in the obligate anaerobic species *Bacteroides*, there was increased growth within the last 12 h. This was observed in all three of the standard microbiota. *Bacteroides* is one of the dominant species in the ascending colon [14].

The complex culture medium used did not contain pure glucose but consisted of potato starch as a carbohydrate source. The use of potato starch as a carbohydrate source may have been the possible cause of the delayed growth of *Bacteroides*. This is because glucose is believed to be a growth-limiting factor in the batch cultivation of *Bacteroides* [31], whereas other bacteria, such as *Bifidobacteria* and *Escherichia*, can break down starch. Due to the requirement that starch must be broken down, there is a delay in the availability of glucose. Therefore, this would explain why the *Bacteroides* only grew in the second half of the cultivation [32,33]. To optimize the cultivation of *Bacteroides*, the addition of glucose to the complex culture medium could be considered in the future. Intestinal bacteria are divided into proteolytic and saccharolytic types. During the metabolism of proteins by proteolytic species, an increase in pH occurs in the colon. In contrast, the saccharolytic types cause a decrease in pH. The reason for this is the formation of SCFAs. Non-digestible complex carbohydrates are the most important substrate source. In the proximal colon, these are fermented by bacteria to SCFAs, H_2_ and CO_2_. 

**Figure 7:**
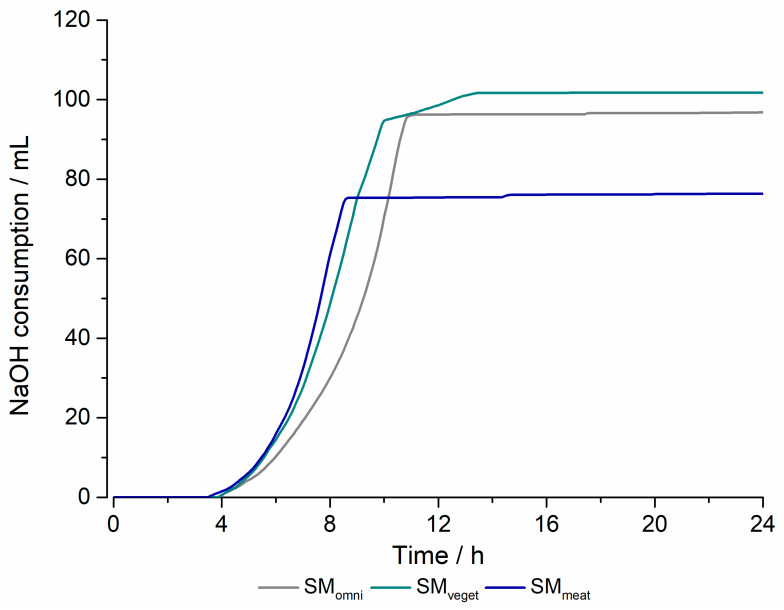
Comparison of base consumption over the cultivation period of 24 h (Biostat^®^ A plus, 2 L complex culture medium, 200 rpm, pH 6.2 (±0.1), 37 °C, *n* = 1).

When cultivating the three different standard microbiota, a different consumption rate of 1 M NaOH was observed. This may be attributed to the standard microbiota having different metabolic activity. The highest consumption rate occurred during the cultivation of SM_veget_, as can be seen in Figure 7. The stool donor of this standard microbiota ate a vegetarian diet. This result may thus be related to the predominantly plant- and carbohydrate-based diet, with acid generation occurring through saccharolytic splitting [14]. Examining the bacterial composition of the SM_veget_ during cultivation, an increase in the bacterial species *Lactobacilli* and *Bifidobacteria* could be noted. This was in contrast to representatives of the proteolytic and saccharolytic types, such as *Enterobacteria*. Similar findings were described in a controlled study by Zimmer et al. In their study, subjects following a vegan or vegetarian diet also had a lower incidence of *Enterobacteria* [13]. The lowering of the pH value due to the increased formation of SCFAs may be the cause of this. This results in the reduced growth of *Enterobacteria* and other pathogenic germs [34]. An Italian study from 2015 also analyzed the influence of diet on the microbiome [15]. The low concentration of lactate-forming bacteria observed in this study was also found in the initial concentration of the SM_veget_. This can be explained by the lack of consumption of fermented foods such as dairy and meat products.

The bacterial composition of the SM_meat_ showed the highest initial concentration of *Enterobacteria*. *Enterobacteria* belong to the species that undergo both saccharolytic and proteolytic nutrition. The donor of SM_meat_ had a predominantly meat-rich diet, which explains the increased occurrence of *Enterobacteria*. The lower base consumption during cultivation may have resulted from the formation of ammonia due to the additional alkalizing effect [35]. SM_omni_ belonged to a donor who had an omnivorous dietary lifestyle. Considering this, the base consumption during cultivation was between the consumption rates of the other two microbiota. After 24 h of cultivation in the Biostat^®^ A plus, the initial differences in the bacterial composition between the standard microbiota could hardly be detected. The similarity of the fermentation products suggested that the complex culture medium had an influence on the composition. On the other hand, the initial starting concentration of bacteria seemed to have a minor influence on the microbiota composition. The availability of the same food sources could serve as an explanation in this context. In a study by Wu et al., the microbiome was shown to change detectably after a 24 h diet. However, this did not transfer to the identity of the enterotype [12]. This supports the assumption that the food sources available influence the bacterial composition of the standard microbiota. Furthermore, in the same study, the consumption of animal protein was detected to have an influence on the abundance of *Bacteroides*. The composition of the complex culture medium, with details provided in Table 1, suggests an alignment with SM_veget_ due to the presence of meat peptone. Moreover, the animal peptone in combination with the increased phosphate content could be the reason for the high proliferation of *Enterobacteria* in all microbiota [36]. Figure 3 shows a comparison of the proportions of anaerobic and aerobic bacterial species over time. In the first 12 h of cultivation, a decreasing trend of anaerobes could be observed in all three microbiota. This could be reconciled with the strong increase in *Enterobacteria* and *Bifidobacteria*. In the second half, the percentage of anaerobic bacteria increased, as well as the concentration of *Bacteroides*. A strong change in the proportions over time could be noted in the SM_omni_. At the beginning, anaerobes constituted the largest proportion, with almost 99%, which was also shown by the high concentrations of *Clostridia* and *Bacteroides*. Consideration should generally be given to the fact that the fecal samples used were a momentary sample, which were undergoing phylogenetic fluctuations [30]. It needs also to be mentioned that the feces do not reflect a direct profile of the populations living in the colon. The more abundant anaerobic species attach to the intestinal mucosa, so identification via feces is limited. However, biopsies of colonic mucosa indicate sufficient similarity to feces to justify such collection of the standard microbiota [37,38,39]. Furthermore, the loss of strictly anaerobic species during sample preparation cannot be eliminated. The determination of the bacterial composition of the standard microbiota used can be assumed to be sufficient for the purposes of the study. In this study, the focus was on the investigation of drug metabolism in the MimiCol^3^.

The previously cultivated standard microbiota were subsequently investigated in the MimiCol^3^ with regard to their influence on sulfasalazine degradation. The physiological parameters used represent the conditions in the ascending colon. The media change was used to simulate the dynamic conditions in the proximal colon. This was used to represent the ileo-caecal reflex, which results in the arrival of new nutrients for the intestinal microbiota and the transport of large volumes [40,41]. This imitation is reflected in the MimiCol^3^ by the fluctuations in the process parameters, as well as the sudden decrease in bacterial density. By adding fresh medium, the bacteria can be kept in an exponential growth phase to achieve the maximum metabolization rate [42]. The increase in bacterial density after each media change can be seen in Figure 6. Continuous gassing with nitrogen is essential to maintain the anaerobic atmosphere. This is particularly relevant for the oxygen-sensitive intestinal bacteria and the *Azoreductase* activity [3,23]. A redox potential below −300 mV was targeted for the anaerobes and could be maintained—indicating reductive, oxygen-free growth and a metabolic environment [27,43]. During the experiments, a pH decrease from the initially measured pH of 7.4, which is the pH value of the Schaedler broth, to the specified value of 6.2 occurred in the vessels between the media changes. The formation of acidic metabolites, such as SCFAs, leads to a drop in the pH value. When combined with the detected increase in OD, this suggests the exponential proliferation of the bacteria [30]. The characteristic intestinal mucosa, with deep crypts, has a decisive influence on the accumulation of certain bacterial species, such as representatives of the *Bacteroides* [14,44]. The MimiCol^3^ is designed to simulate certain aspects of the ascending colon, such as bacterial colonization, temperature and pH [45]. With the defined test conditions in the MimiCol^3^, a compromise between the adequate reproduction of the complex physiological conditions and an appropriate design of the in vitro tool could be created.

**Figure 8:**
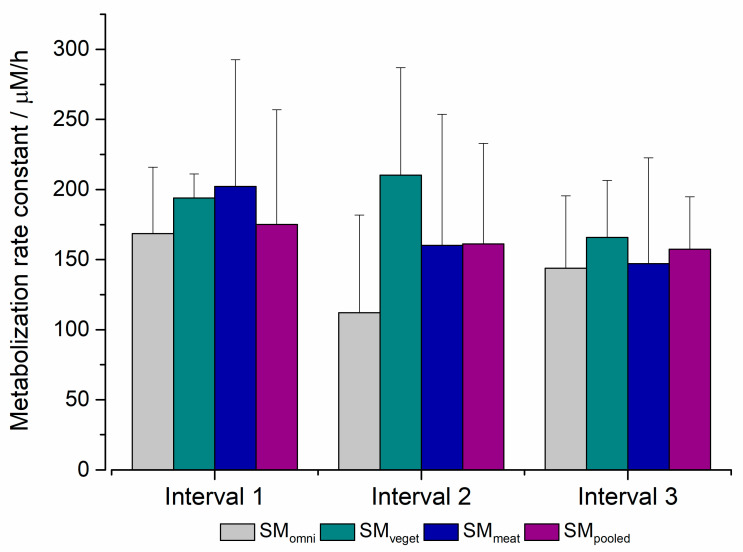
Metabolization rate constants of sulfasalazine in the MimiCol^3^ (150 mL Schaedler broth, 100 rpm, pH 6.2 (±0.25), 37 °C, *n* = 4 for SM_omni_, SM_veget_ and SM_meat_, *n* = 3 for SM_pooled_ (mean + SD)).

To be able to characterize the metabolization performance of the different standard microbiota, sulfasalazine degradation was compared at given intervals. The reductive cleavage of the azo bond of sulfasalazine into the metabolites 5-aminosalicylic acid and sulfapyridine caused by *Azoreductases* was quantified by UV–VIS measurement [46]. Only the decrease in sulfasalazine was detected; metabolites being generated were not evaluated. As in the Biostat^®^ A plus, bacterial growth was monitored based on the OD. With an increase in OD, a reduction in the amount of drug present in the vessel could be detected. By calculating the correlation coefficients, an inverse linear correlation between bacterial growth and drug content was found for each microbiota. The measured OD absorption data at comparable time intervals to the Biostat^®^ A plus were higher. This could be explained by the Schaedler broth used as the medium, since the presence of glucose positively influenced the growth of the genera of *Bifidobacteria* and *Bacteroides* [47]. From Figure 8, differences in the metabolization rate between the bacterial culture can be noted. 

The acquired data on the metabolization performance varied widely for each standard microbiota. Compared to the other microbiota, SM_omni_ differed most markedly, showing the lowest absorption values as well as the lowest metabolization rates. The differences between SM_meat_ and SM_pooled_ were the lowest. In the SM_meat_, most of the drug was metabolized in the first interval, with more than 200 µM/h. The same applied to SM_omni_, where only 170 µM/h was metabolized. The metabolization rate of the SM_pooled_ decreased slightly over time. For SM_veget_, the highest drug metabolization was noted in the second interval. However, the differences between the different standard microbiota were less pronounced than expected. From Figure 8, it becomes clear that the metabolic rates from the different microbiota were comparable. This can be explained by the fact that the compositions of the standard microbiota were found to be quite similar and their differences were lost during cultivation in the Biostat^®^ A plus. Thus, there was already a limitation during cultivation that influenced the further course of the experiments in the MimiCol^3^. To prevent this loss in the future, different media should be considered when cultivating the microbiota. The diversity of the bacterial composition, then, should be maintained based on different diets during cultivation. By maintaining this diversity, potential influences on drug degradation can be highlighted more clearly.

Following the bacterial growth of the individual standard microbiota, shown in Figure 4, it can be compared with the drug degradation. However, the slightly increased bacterial occurrence of the SM_veget_ had a weaker effect on the degradation of the drug. While, in the second interval, the highest metabolic efficiency was achieved, the difference was already reduced in the third interval. Since the measurement of the OD is only the determination of turbidity, the susceptibility to interference should be considered in the evaluation. Although these measurements are indicative of bacterial growth, they should be viewed critically because they are a dimensionless quantity. To accurately characterize bacterial growth, CFU samples from the different test days and standard microbiota were plated out and counted.

The coefficients of variation showed differences in the determination of OD, especially immediately after the media changes. However, this was different for the degradation of sulfasalazine. In this case, there was scatter in the OD of the microbiota at the end of a change interval, which resulted in a difference in the maximum amount of drug degraded. The differences may be due to the remaining 15 mL of media in the vessel at the time of the media change. Due to the different residual volumes remaining in the vessel, there may have been both a difference in bacterial density and a difference in the composition of the remaining bacteria for the following time intervals, which may have affected the degradation of the drug. The scatter of the OD and sulfasalazine degradation shown in Figure 4 and Figure 5 can be explained by this. For this reason, the variable growth and metabolic activity of the bacteria caused by biological fluctuations/changes must also be considered. 

According to the literature, the increased metabolization constant in the first interval in SM_meat_ could be associated with the increased presence of *Azoreductase*-producing bacteria of the genera *Enterobacteria*, *Clostridia* and *Bacteroides* [23,48]. Comparing this with the evaluated CFU samples in Figure 6, the occurrence of *Enterobacteria* was highest in the SM_pooled_, with SM_veget_ containing the largest proportion during the first interval. This assumption is also not valid for *Clostridia*. However, it should be mentioned that this characterization is representative of a single sample and a detailed characterization of the species by plating on agar media was not possible. As already stated, due consideration should be given to the fact that there was consistency during the cultivation of different standard microbiota. No significant differences in the standard microbiota could be detected during the experiments, except for their source of origin. The bacterial growth as well as drug degradation recorded in the SM_pooled_ represent, as expected, a mixed form of the other microbiota. The different metabolization rates over the experimental period may also relate to the correlation between the growth stage and metabolization performance, described by Maier [42]. In the study by Kellow et al., the rate of metabolism of sulfasalazine was used to characterize the intestinal transit time. During the study by Kellow et al., flooding of the metabolite sulfapyridine with a plasma concentration of 0.1–0.6 µg/mL was detected within 1 to 6 min after the injection of 2 g sulfasalazine into the cecum [49]. This suggests the bacterial degradation of sulfasalazine. In the MimiCol^3^, the reduction of sulfasalazine could be detected approximately 25 min after injection. The slower metabolization may have been related to the absence of the intestinal epithelium in the vessel, which is associated with the growth and metabolism of some bacterial species [44]. Nevertheless, the data collected indicated the sufficient presence of *Azoreductases* and suggested biorelevant drug metabolism in the MimiCol^3^. Comparing the metabolization rate data collected in this study with those from the publication by Beeck et al., these rates are lower overall. The comparison of the metabolization rates in the single intervals also differed. In the newly obtained data, the highest conversions were recorded in the first interval, while, in the series by Beeck et al., this was observed in the second interval [28].

During the experiments, it was found that deviations occurred in the third vessel of the MimiCol^3^. A reduction in bacterial growth and sulfasalazine degradation occurred, which was independent of the standard microbiota used. When analyzing the error, a complication in the base pump control was identified. The required base volume to maintain the pH value at 6.2 ± 0.25 could therefore not be continuously supplied. Studies on the pH dependence of the *Azoreductases* show the stability of the enzymes over a pH range of 5–7. However, the optimum activity lies in the physiological range. Considering this, a deviation in catalytic performance may have occurred in the third vessel. This would explain the reduced drug degradation. Furthermore, the sensitivity of *Enterobacteria* to low pH values is described in the literature [34]. Since *Enterobacteria* are *Azoreductase* producers, this could have been another cause of the reduced degradation. Another aspect of the error in the analysis was deviations in the volume in vessel 3. This was most marked when the medium was removed. As a result, the bacterial density was reduced. Thus, the reduced bacterial growth had an additional negative effect on the metabolism of sulfasalazine. The problems described above need to be resolved in the future so that the model can be further optimized.

Although the number of stool donors was kept very small (*n* = 1) in this series of experiments, differences in the composition of the microbiota depending on the diet could be identified at the beginning of the cultivation. However, the diversity of the individual stool microbiota was already lost during the batch cultivation. Nevertheless, in the simplified setup of MimiCol^3^, signs of different metabolic rates could be detected. However, the results showed that the complex culture medium used for cultivation played a crucial role and it was not targeted in this study. The previous dietary habits of the donors became less important as the diversity was lost during cultivation. 

It is important to minimize this loss in order to better investigate the possible effects of diet on drug metabolism. For this purpose, the use of different cultivation media for individual microbiota might be considered. Another possibility for individual experiments would be the use of a freshly generated stool sample in the model. However, this would impede the repetition of the experiments under similar conditions. The influence of the microbiota on drug metabolism cannot be neglected as it can contribute significantly to the success of therapy, which needs to be further investigated.

## 4. Materials and Methods

### 4.1. Materials

Azulfidine tablets (Pfizer, New York City, NY, USA) containing 500 mg of sulfasalazine were obtained from the university’s hospital pharmacy. Commercially available sulfasalazine, suitable for analytical experiments, was purchased from Fluka^®^ Analytical (Seelze, Germany). Schaedler broth was purchased from Carl Roth (Karlsruhe, Germany) as a dry substance and reconstituted according to the manufacturer’s specifications. Glycerol Rotipuran 99.5% was purchased from Caesar & Loretz GmbH (Hilden, Germany) and diluted to obtain a concentration of 20%. The individual components of the complex culture medium were purchased from Sigma-Aldrich (St. Louis, MO, USA), VWR International (Radnor, PA, USA) and Carl Roth. The various agar media and the required supplements were obtained from Sigma-Aldrich/Fluka and Carl Roth, while the components of the peptone water were acquired from Carl Roth, Fluka and BDH Prolabo/VWR International. Sterile water for injection (Ph. Eur. 11.2) served as a solvent in all cases.

### 4.2. Methods

#### 4.2.1. Preparation of Standard Microbiota

The stool samples were taken from healthy volunteers (1 male, 2 females, age 26–29 years, body mass index 18–35 kg/m^2^). The volunteers were recruited based on their different lifestyles in terms of their dietary choices. Standard microbiota based on an omnivorous (SM_omni_), a vegetarian (SM_veget_) and a meat-rich (SM_meat_) diet were compared. Due to time constraints, it was decided to use *n* = 1 initially, so that a trend for the influence of diet on the degradation of sulfasalazine in MimiCol^3^ could at least be determined. The recruitment of volunteers and the collection of their feces was approved by the ethics committee of University Medicine Greifswald (BB 009/22). Written informed consent was obtained from all volunteers prior to sample collection.

For the generation of each different standard microbiota, 1.00 g fresh feces was suspended in 40 mL peptone water and allowed to rest for 5 min. After sedimentation, the supernatant was then used for the cultivation of the standard microbiota in the batch fermenter Biostat^®^ A plus (Sartorius stedim Biotech, Göttingen, Germany). The following settings were used in the Biostat^®^ A plus to ensure optimal conditions for the cultivation of the standard microbiota. The vessel, with a capacity of 2000 mL and an integrated stirring system that was programmed to 200 rpm to ensure continuous mixing of the medium, was filled with the complex culture medium and kept at a temperature of 37 °C. The details of the composition can be found in Table 1. 

The pH of the complex culture medium was set to 6.2 (±0.1). To obtain anaerobic conditions during the cultivation, the vessel was flushed with nitrogen 1 h prior to the start of the process. The cultivation process continued for 24 h, with 5 mL of sample taken every 4 h. This sample was measured for optical density (OD) to monitor the bacterial growth. Then, 200 µL of the sample was diluted with 1800 µL distilled water and was analyzed with UV–VIS spectroscopy (Cary 50 Scan UV Visible Spectrophotometer, Varian Inc., Palo Alto, CA, USA) at 600 nm in quartz cuvettes (path length = 1 cm). The sample at time 0.083 h was used as a reference to compare the bacterial growth. After 24 h, the entire medium was removed from the vessel and processed as follows. First, 25 mL of bacterial culture was diluted with 25 mL glycerol 20%, stored at −20 °C for 24 h and then transferred to −80 °C.

For the following studies of the bacterial composition, CFU samples were prepared as follows. First, 300 µL samples were mixed with 300 µL glycerol 20% and vortexed. Subsequently, these were stored at −20 °C for 24 h and transferred to −80 °C until used for characterization.

#### 4.2.2. Characterization of Standard Microbiota

Different agar plates were prepared for the characterization of CFU samples. Table 2 provides the agar media used for the different bacteria species. The plating was performed under aseptic conditions. Samples from the time points 0, 12 and 24 h were plated out. The cryo samples were diluted with sterilized peptone water according to the following dilution series. The dilution series was performed in steps of ten from 1 to 1 million. For each dilution, 20 µL was plated on each agar medium. The inoculated agar plates were incubated for two days under aerobic conditions (INB 400, Memmert GmbH, Schwabach, Germany) at 33 °C or under anaerobic conditions (Whitley A35 Anaerobic Workstation, Don Whitley Scientific, Bingley, UK) at 37 °C for five days. The CFU were determined by counting. The plate sections with the highest concentration and greatest bacterial growth, ranging from 3 to 90 colonies, were used for counting. CFU/mL was calculated using the following Equation (1). A sample volume of 20 µL was used (‘50’) and the sample was mixed with glycerol in a ratio of 1:2 (‘2’).

(1) $$\frac{CFU}{mL}=CFU\times dilution\;factor\times 50\times 2$$

#### 4.2.3. Experimental Procedure

**Figure 9:**
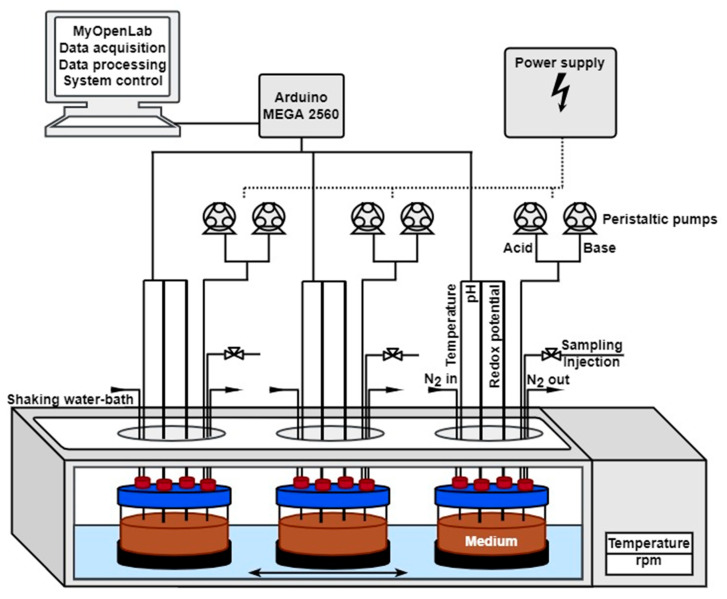
Schematic representation of the MimiCol^3^ by Beeck et al. [28].

The subsequent experiments on sulfasalazine degradation were carried out in the MimiCol^3^, an in vitro model that reflects the conditions of the ascending colon. A more detailed methodological description can be found in a previous publication from Beeck et al. [28]. A schematic overview of the MimiCol^3^ is shown in Figure 9.

In brief, the model consists of three glass vessels with a capacity of 250 mL each. A pH control of 6.2 (±0.25) is set for the medium, with an initial pH of 7.4. Due to bacterial metabolic processes, pH drift over time can occur. Therefore, two programmable peristaltic pumps are used to keep the pH in the desired range by titrating with 1 M HCl and 1 M NaOH solution. Once the pH drops below 6.4, the pH control is activated and deactivated during the media change. To ensure mixing of the medium, the vessels are placed in a shaking water bath (SW22 model, JULABO GmbH, Seelbach, Germany) at 37 °C with the shaking motion set to 100 rpm. To create an anaerobic atmosphere, the vessels are continuously gassed with nitrogen. For control purposes, a pH and redox electrode are inserted via the lids of the vessels, as well as a temperature sensor. The measured values are recorded and controlled using the software *MyOpenLab*^®^ (version 3.11.0). The data recording for vessel 1 occurs digitally. The data for vessel 2 and 3 are recorded manually. An illustration of the evaluated process for the parameters can be obtained from a publication by Beeck et al. [28].

The experimental conditions were based on those of the MimiCol by Beeck et al. [45]. The inocula required for the experimental series were prepared on the day of the experiment as follows. The samples stored at −80 °C were thawed in a water bath at 37 °C for approximately 20–30 min. The thawed samples were centrifuged at 6000 rpm for 6 min and the supernatant discarded. The bacterial pellet was resuspended in 10 mL Schaedler broth and injected into the appropriate vessel at the start of the experiment. For the series of experiments with the pooled standard microbiota (SM_pooled_), the three thawed stool samples were homogenized in a flask and then divided equally among sampling tubes. They were centrifuged at 6000 rpm for 6 min, and the bacterial pellet was resuspended in Schaedler broth before they were injected into the vessels. After gassing with nitrogen for 1 h, the standard microbiota was injected at the beginning of the experiment. The bacteria were incubated over a period of 3 h. Media changes were performed after 3, 5 and 7 h to ensure bacterial growth by adding fresh nutrients. The media change was performed in the ratio 90:10, leaving a residual volume of 15 mL in each vessel. This was followed by the addition of 135 mL pre-tempered Schaedler broth, which contained 30 mg of powdered Azulfidine^®^ tablets (23.5 mg of sulfasalazine). 

Samples of 5 mL were taken at 30 min intervals to determine bacterial growth by OD and to determine the metabolism of sulfasalazine by UV–VIS spectroscopy. The removed medium was not replaced at the time of sample collection in this experimental setup. To determine the drug concentration, 2000 µL samples were centrifuged at 14,500 rpm for 6 min (MiniSpin^®^ plus, Eppendorf AG, Hamburg, Germany). Then, 200 µL supernatant was taken and diluted with 1800 µL 0.1 M NaOH and analyzed photometrically at 450 nm in quartz cuvettes (path length = 1 cm). Due to the bathochromic absorption shift of sulfasalazine at pH values above pH 10, dilution with 0.1 M NaOH was performed. This reduced the background noise of the Schaedler broth. Concentrations were calculated using the calibration curve of a sulfasalazine analytical standard in 0.1 M NaOH, as described in Section 4.2.4. Metabolization constants were calculated as follows: (i) the concentration in µg/mL was plotted against time in h; (ii) the negative slope of the linear section of the curve of the last three samples from an interval between media changes was determined as zero-order kinetics was assumed; and (iii) it was converted into µM/h via the molar mass of sulfasalazine (398.38 g/mol).

To determine the OD, a 200 µL sample was diluted with 1800 µL distilled water. Samples were analyzed photometrically as previously described. Here, 200 µL samples from 0.083 h and 1800 µL distilled water were measured as a blank sample. To analyze bacterial growth, CFU samples were taken hourly and after the medium was changed. The samples were prepared and stored as described in Section 4.2.1.

To obtain representative results, an alternating distribution of the different standard microbiota among the three vessels was applied in the experiments. The purpose of rotating the vessels was to minimize any bias in the results caused by the model. This was done to ensure that the results were as representative as possible. In this way, the same initial situation was created for all the microbiota investigated. For the series of experiments, both the individual standard microbiota and a pooled standard microbiota were used. Table 3 shows the assignments of the microbiota to each vessel of the in vitro model for each experiment.

#### 4.2.4. Preparation of Calibration Curves

A stock solution with a concentration of 0.4 mg/mL of the analytical sulfasalazine standard was prepared to calculate the sulfasalazine concentration. From this stock solution, 20, 40, 60, 80, 100 and 120 µL were diluted to 2 mL with 0.1 M NaOH and measured photometrically at 450 nm. The absorption was plotted against the concentration of sulfasalazine in µg/mL, showing a good linear correlation between 4 and 24 µg/mL. Therefore, a coefficient of determination of R^2^ > 0.998 was considered as an acceptance criterion. A calibration curve was freshly prepared on each day of the experiment.

## 5. Conclusions

The study of the biologically relevant metabolism of sulfasalazine by the gut microbiota was successful in the MimiCol^3^. To ensure that as many bacteria as possible were available for the experiments, different diets were considered when recruiting fecal donors. The focus was on omnivorous, vegetarian and meat-rich diets. However, when the stool samples were cultured in the Biostat^®^ A plus, the loss of the above-mentioned properties was observed, and thus a decrease in the diversity of the bacterial microbiota among themselves, which was also reflected in the results of sulfasalazine metabolism. Looking at the results of sulfasalazine degradation, the degradation of the active ingredient occurred during all experiments. This is consistent with the exponential growth of bacteria due to the conditions in the MimiCol^3^. Despite the simplicity of the model, a reproducible in vivo study environment for the cultivation of colonic-relevant bacterial species has been established. Despite the reduction in the desired diversity of the bacterial composition, differences in drug degradation were observed. The design of the MimiCol^3^, consisting of three vessels, allowed experiments to be run in parallel, resulting in a higher yield of results. It was found that the variability within one day was lower than in experiments carried out on different days. The in vitro model MimiCol^3^ can be further optimized through a series of experiments with substances for which metabolism by the colonic microbiome has already been demonstrated. In the future, it should be possible to make further statements about the degradation of known active substances, as well as performing initial assessments of new substances.

## Figures and Tables

**Table 1:** Composition of the complex culture medium (according to Minekus et al. [50]).

Solution	Compound	Concentration g/L
1	Dipotassium hydrogen phosphate	2.50
	Sodium chloride	4.50
	Iron(II)sulfate heptahydrate	0.005
2	Calcium chloride dihydrate	0.45
	Magnesium sulfate heptahydrate	0.50
3	Pectin (apple)	0.60
	Xylan (beech)	0.60
	Arabinogalactan	0.60
	Amylopectin (corn)	0.60
	Starch (potato)	5.00
4	L-cysteine	0.40
5	Meat peptones	3.00
	Casein (cow’s milk)	3.00
	Ox bile	0.05
6	Polysorbate 80	2.00
7	Hemin	0.01

**Table 2:** Assignment of different agar media for the detection of bacterial sub-populations.

Agar Media	Bacteria Species
Tryptic soy agar (TSA/CASO)	Total aerobic
MacConkey agar	*Enterobacteriaceae*
Man, Rogosa and Sharpe (MRS) agar	*Lactobacilli*
Modified reinforced clostridial medium	*Clostridia*
Bifidus Selective Medium (BSM) agar	*Bifidobacteria*
Modified Schaedler agar	*Bacteroides*
Schaedler agar	Total anaerobes

**Table 3:** Different distributions of the standard microbiota in each of the three vessels of the MimiCol^3^.

Experiment	Vessel 1	Vessel 2	Vessel 3
1	SM_omni_	SM_veget_	SM_meat_
2	SM_meat_	SM_omni_	SM_veget_
3	SM_veget_	SM_meat_	SM_omni_
4	SM_pooled_	SM_pooled_	SM_pooled_
5	SM_omni_	SM_veget_	SM_meat_

## Data Availability

The data presented in this study are available at the University of Greifswald’s storage system.
